# Application of Evidence-Based Methods to Construct Mechanism-Driven Chemical Assessment Frameworks

**DOI:** 10.14573/altex.2202141

**Published:** 2022-03-01

**Authors:** Sebastian Hoffmann, Elisa Aiassa, Michelle Angrish, Claire Beausoleil, Frederic Y. Bois, Laura Ciccolallo, Peter S. Craig, Rob B. M. de Vries, Jean Lou C. M. Dorne, Ingrid L. Druwe, Stephen W. Edwards, Chantra Eskes, Marios Georgiadis, Thomas Hartung, Aude Kienzler, Elisabeth A. Kristjansson, Juleen Lam, Laura Martino, Bette Meek, Rebecca L. Morgan, Irene Munoz-Guajardo, Pamela D. Noyes, Elena Parmelli, Aldert Piersma, Andrew Rooney, Emily Sena, Kristie Sullivan, José Tarazona, Andrea Terron, Kris Thayer, Jan Turner, Jos Verbeek, Didier Verloo, Mathieu Vinken, Sean Watford, Paul Whaley, Daniele Wikoff, Kate Willett, Katya Tsaioun

**Affiliations:** 1 Evidence-based Toxicology Collaboration (EBTC) at Johns Hopkins Bloomberg School of Public Health, Baltimore, MD, USA;; 2 European Food Safety Authority (EFSA), Parma, Italy;; 3 United States Environmental Protection Agency, Office of Research and Development, Center for Public Health and Environmental Assessment, Research Triangle Park, NC, USA;; 4 ANSES, Risk Assessment Department, Maisons-Alfort, France;; 5 Certara UK, Simcyp Division, Sheffield, UK;; 6 Durham University, Durham, UK;; 7 RTI International, Research Triangle Park, NC, USA;; 8 SeCAM, Magliaso, Switzerland;; 9 current affiliation: European Food Safety Authority (EFSA), Parma, Italy;; 10 CAAT-Europe, University of Konstanz, Konstanz, Germany;; 11 European Commission, Joint Research Centre, Ispra, Italy;; 12 University of Ottawa, Ottawa, Canada;; 13 California State University, East Bay, CA, USA;; 14 Department of Health Research Methods, Evidence and Impact, McMaster University, Hamilton, ON, Canada;; 15 Centre for Health Protection (RIVM), Bilthoven, the Netherlands;; 16 Division of the National Toxicology Program, National Institute of Environmental Health Sciences, Research Triangle Park, NC, USA;; 17 University of Edinburgh, Edinburgh, UK;; 18 Physicians Committee for Responsible Medicine, Washington, DC, USA;; 19 Safer Medicines Trust, Kingsbridge, UK;; 20 University of Eastern Finland, Kuopio, Finland;; 21 Vrije Universiteit Brussel, Brussels, Belgium;; 22 Booz Allen Hamilton, Rockville, MD, USA;; 23 Lancaster Environment Centre, Lancaster University, Lancaster, UK;; 24 ToxStrategies, Asheville, NC, USA;; 25 Humane Society International, Washington, DC, USA

## Abstract

The workshop titled “Application of evidence-based methods to construct mechanism-driven chemical assessment frameworks” was co-organized by the Evidence-based Toxicology Collaboration and the European Food Safety Authority (EFSA) and hosted by EFSA at its headquarters in Parma, Italy on October 2 and 3, 2019. The goal was to explore integration of systematic review with mechanistic evidence evaluation. Participants were invited to work on concrete products to advance the exploration of how evidence-based approaches can support the development and application of adverse outcome pathways (AOP) in chemical risk assessment. The workshop discussions were centered around three related themes: 1) assessing certainty in AOPs, 2) literature-based AOP development, and 3) integrating certainty in AOPs and non-animal evidence into decision frameworks. Several challenges, mostly related to methodology, were identified and largely determined the workshop recommendations. The workshop recommendations included the comparison and potential alignment of processes used to develop AOP and systematic review methodology, including the translation of vocabulary of evidence-based methods to AOP and *vice versa*, the development and improvement of evidence mapping and text mining methods and tools, as well as a call for a fundamental change in chemical risk and uncertainty assessment methodology if to be conducted based on AOPs and new approach methodologies (NAM). The usefulness of evidence-based approaches for mechanism-based chemical risk assessments was stressed, particularly the potential contribution of the rigor and transparency inherent to such approaches in building stakeholders’ trust for implementation of NAM evidence and AOPs into chemical risk assessment.

## 1 Introduction

Systematic, evidence-based methods to review and assess scientific information and adverse outcome pathways (AOPs), a construct to structure biological events resulting in adverse effects, are two methodological approaches intended to advance toxicological research and decision-making in the 21^st^ century. Systematic approaches comprise evidence synthesis methods, such as systematic reviews (SR) and evidence maps (Hoffmann et al., 2017; Wolffe et al., 2019), but also tools for specific tasks, e.g., for the critical appraisal of studies or the assessment of the certainty in a body of evidence (Lam et al., 2014; Samuel et al., 2016; Dishaw et al., 2020). Such approaches have also been suggested as an approach to the validation of new approach methods (Hartung et al., 2013). The AOP framework is intended to describe and interlink biological/toxicological key events (KE) leading to an adverse outcome. It provides a construct of levels of biological organization with increasing complexity, starting from molecular initiating events (MIE) to adverse outcomes (AO) manifested in populations of organisms, on which to map our knowledge of toxicological mechanisms and of their causal connections. The construct is aimed at facilitating the integration of *in vitro* and *in vivo* toxicological data, as well as epidemiological results, to inform regulatory decisions and to guide the development of new approach methodologies (NAM) (Ankley et al., 2010; Villeneuve et al., 2014; Tollefsen et al., 2014; OECD, 2016; Leist et al., 2017; Ankley and Edwards, 2018).

At the Grading of Recommendations Assessment, Development and Evaluation (GRADE) Working Group satellite workshop, held on June 12, 2019, in Hamilton, Canada, it was discussed how systematic review methods and AOP concepts can be combined to develop and use NAMs for predicting the toxicity of chemical substances to humans in an evidence-based manner, i.e., transparently, objectively, reproducibly, and consistently (De Vries et al., 2021). This workshop, organized by the Evidence-based Toxicology Collaboration at Johns Hopkins Bloomberg School of Public Health (EBTC), brought together researchers of the various stakeholders who are actively exploring the synergistic potential of systematic review methodology and AOP, and GRADE Working Group members experienced in the use of evidence-based approaches in potentially similar clinical applications. Key workshop results were:

1. The AOP framework is a promising approach to assemble mechanistic evidence for risk assessment;
2. evidence-based methodology, including systematic reviews, has the potential to facilitate development of AOPs; and
3. communication obstacles need to be overcome to optimize collaboration of the two fields.

To further foster this collaboration, a follow-up workshop, summarized here, was organized, also by the EBTC, to deepen the discussions and work on concrete products to advance the field. The workshop was held at the European Food Safety Authority (EFSA), Parma, Italy, on October 2 and 3, 2019, hosted by EFSA and the EBTC at Johns Hopkins Bloomberg School of Public Health (EBTC).

The workshop was opened with a welcome by Bernhard Url, the executive director of EFSA. He briefly introduced EFSA, stressing that its scope of work is risk assessment and that the risk is managed by the European Commission, Member State authorities and the European Parliament. He then made clear that the foundation of transparency has been the basis for the productive collaboration between EFSA and EBTC. Transparency and impartiality, and the lack and perceived lack thereof, has been a fundamental challenge for risk assessors, e.g., in EFSA since its foundation. Debates on these topics peaked over the years around several substances, most recently on bisphenol A and glyphosate, resulting in a loss of decision maker trust in the underlying science. EFSA considers interested experts as essential for their risk assessment tasks, as long as those interests do not constitute potential conflicts of interest in a given context. Therefore, conflicts of interest are understood as situation-specific and not expert-specific.

Transparency of data and how data are appraised and integrated has become a focus in EFSA, as it provides the means for EFSA to be held accountable for their work. In particular, a logical framework to make processes such as weighting of evidence (WoE) fully transparent is essential. As an example of how EFSA could further improve its processes, he mentioned that instead of considering the quality of studies that comply with Good Laboratory Practices (GLP) as high by default, a detailed assessment of studies could be conducted. Ultimately, improved processes will allow us to make evidence-based decisions and, more importantly, to demonstrate impartiality. Behaving in such a trustworthy manner is aimed at increasing social trust. In addition, EFSA intends to create trust, even if assessment outcomes conflict with political values, by engaging with civil society. Scientists should look beyond evidence, which, in doubt, will not override societal values, and consider how they can contribute to the creation of societal trust, e.g., through educational activities. To achieve this goal of trustworthiness, evidence-based decision-making is needed, optimally based on a collaborative global risk assessment network with a common understanding of scientific methodology.

Katya Tsaioun, director of the EBTC at Johns Hopkins Bloomberg School of Public Health, and Rob de Vries (EBTC) introduced the participants to the workshop topic. Starting with a joint colloquium of EFSA and EBTC in 2017 (summarized in EFSA and EBTC, 2018), the issue of assessing and integrating evidence relevant for the toxicological mechanisms of chemicals in a systematic and evidence-based manner, particularly when described as AOPs, has been attracting considerable attention. As AOPs are based on existing knowledge, systematic evidence-based approaches promise to be a potentially useful methodological approach, if appropriately adapted, to ensure comprehensiveness of the evidence used in the construction of AOPs and to standardize AOP development across applications.

A major potential strength of AOPs is in their holistic view of the relevance of individual pieces of evidence to a health assessment. By using AOPs, studies that may appear only indirectly relevant to assessing health risks from exposures can be aggregated into a connected body of evidence. In making these connections, the evidence base can be considered as a whole.

To bring researchers interested in the topic together, the EBTC and the GRADE Working Group organized a workshop on “Applying Evidence-Based Methods to the Development and Use of Adverse Outcome Pathways” (De Vries et al., 2021). This workshop preceded the annual GRADE meeting in order to attract and benefit from participants with clinical and medical expertise and experience in using the GRADE approach to evidence assessment, which is established for assessing certainty in the evidence within a systematic review (Guyatt et al., 2011). Widely used in healthcare and public health systematic reviews, this approach is now increasingly being applied in the context of environmental health and chemical assessments (Morgan et al., 2016). This is because much of the environmental health evidence base is only indirectly related to the human contexts of concern. For example, the best available evidence to address a human health concern may only be found in a body of indirect evidence, such as animal studies or *in vitro* studies, i.e., not generated in the population of interest. The AOPs framework to structure and integrate evidence potentially allows for fuller use of indirect evidence in a systematic review and may increase certainty of the relationship between the potentially indirect evidence base and the elements of the question for which it is informative. AOPs are therefore of strong interest when applying GRADE to SRs of studies of environmental exposures.

The key messages from that EBTC/GRADE workshop were:

- Systematic and transparent assembly of literature-based evidence could support the assessment of the certainty in AOPs.
- Automated text mining and machine learning tools can facilitate systematic literature review and mapping of evidence to the AOP framework, facilitating more transparent AOP development.
- The GRADE framework for assessing the certainty in the body of evidence is likely applicable to AOP certainty assessment.
- Collaboration is key for better mutual understanding between the AOP and SR communities, creating a common vocabulary, and for collectively advancing the fields of AOP development and systematic review.
- Classical risk of bias frameworks do not fit all types of NAMs, e.g., as the application and impact of bias-reducing factors such as randomization and blinding is not straightforward.
- New approaches are needed to assess the certainty in individual decisions, which is a function of confidence in the AOP and NAM data used to inform the AOP KE and key event relationships (KER)

From these messages, three themes were selected for the workshop and are summarized here:

1. Assessing certainty in AOPs
2. Literature-based AOP development
3. Integrating certainty in AOPs and NAM evidence into decision frameworks

Participants were assigned to one of the three themes at the start of the workshop. Per theme, two theme-specific presentations were given in plenary sessions. These were intended to stimulate discussion in the theme groups in break-out sessions, for which the groups were charged with specific questions. In addition to the theme-specific presentations, two more general plenary lectures were given.

Rather than following the chronological sequence of the agenda, this report is structured around the topics addressed. Therefore, the two lectures are summarized first, followed by the three themes, for each of which the theme-specific presentations and break-out session results are presented.

## 2 Introduction to evidence-based methods and mechanistic evidence including AOPs

In the first lecture, Juleen Lam introduced the audience to evidence-based methods and mechanistic evidence including AOPs. She started by stressing that decision-making to prevent harmful exposure in public health relies on high-quality scientific evidence. Robust methods to synthesize links between environmental exposures and harmful health effects are necessary to inform public policy and prevent harm. However, environmental health decisions have historically relied on expert-based narrative reviews to summarize the collection of scientific information (Woodruff and Sutton, 2014). Narrative reviews have drawn criticisms for several reasons, namely lack of transparency and rigor, limited ease by which new literature can update conclusions, and restricted utility in making resource and investment decisions, in supporting and justifying policy decisions, and in contributing to improvement of the scientific evidence base. With mounting evidence that harmful chemical exposures are increasing and contributing to adverse health outcomes, it is apparent that a rigorous framework for decision makers to base policies and actions on the best available scientific information is vital to ensure adequate public health protection.

Systematic review methods, defined as a review of literature focused on a specific question that uses explicit, pre-specified methods to identify, select, assess, and synthesize scientific evidence (Institute of Medicine, 2011), have been proposed as a potential solution to address many of these challenges. Prominent systematic review methods such as those developed by the Cochrane Collaboration (Higgins et al., 2019) and GRADE (Guyatt et al., 2008) are regularly employed in healthcare to inform decisions that lead to cost savings and improved health outcomes (Fox, 2010).

The integration of systematic review methods within the field of environmental health is relatively recent but has been under development for the past decade. Several systematic review frameworks currently exist, including those that have been applied to several case studies in the environmental health field (Woodruff and Sutton, 2014; Rooney et al., 2014; NRC, 2014). Despite minor differences in the application of these approaches, overarching commonalities exist in the fundamental steps of a systematic review: 1) specify a research question; 2) prepare a protocol; 3) search for evidence; 4) select evidence; 5) extract data; 6) synthesize data; 7) assess risk of bias of individual studies; 8) rate the overall certainty in the body of evidence in addressing the research question.

Numerous proof-of-concept case studies demonstrate the application of systematic review for environmental health questions of concern (Lam et al., 2014; Rooney et al., 2015; Vesterinen et al., 2015; Johnson et al., 2016; Lam et al., 2016, 2017; NRC, 2017; NTP, 2016, 2019). These case studies illustrate the evaluation and integration of experimental animal and observational human evidence to establish bottom line conclusions regarding the overall strength of evidence in support of potential human health effects. However, these frameworks offer limited guidance for considering *in vitro* data (i.e., assays based on human cells or human cell constituents that measure effects on toxicity pathways relevant to adverse human health outcomes (Judson et al., 2010)). These data can have advantages over traditional evidence streams, including the ability to gain insight on lower dose exposures, early markers on the toxicity pathway, and human cell-based models that increase human predictivity potential (Bal-Price and Meek, 2017).

With increasing efforts to use *in vitro* data in environmental chemical assessments (Knight, 2008; Kavlock et al., 2009; Abbott, 2009; NRC, 2007), a critical need remains to establish frameworks to integrate this type of evidence stream into the systematic review process. In particular, established frameworks to evaluate the risk of bias (i.e., internal validity) as well as strength, quality, or confidence in *in vitro* data are lacking. In regard to risk of bias – a measure of whether the design or conduct of an individual study compromises credibility of the reported results (Guyatt et al., 2011; Institute of Medicine, 2011; Viswanathan et al., 2012) – several tools exist to evaluate the reporting or to critically appraise the conduct of primary *in vitro* studies (Klimisch et al., 1997; Krithikadatta et al., 2014; Schneider et al., 2009; Beronius et al., 2018). However, the adequacy of some of these tools has been called into question (Ågerstrand et al., 2011a,b). To date, none have been evaluated, tested, or incorporated into a systematic review framework, although efforts are currently underway to do so (De Vries and Whaley, 2018).

In considering approaches to integrate *in vitro* data with systematic review frameworks, several organizational mechanisms currently exist that may be considered. For instance, modes of action (MoA) and AOP are organizational frameworks that describe chemical or biological KE that result from perturbations from a chemical stressor (Bal-Price et al., 2017). Key characteristics are another organizational approach for *in vitro* data developed for a variety of human health outcomes – carcinogenicity (Smith et al., 2016), male reproduction (Arzuaga et al., 2019), female reproduction (Luderer et al., 2019), and endocrine disruption (La Merrill et al., 2019). These may be helpful as a starting point to identify, organize, and analyze *in vitro* data, and potentially as a way to streamline these data into systematic review processes.

With the pivotal shift of environmental health risk assessment towards increased utilization of *in vitro* data, the need to integrate these data with systematic review methodology for chemical assessment is apparent. However, there remains a critical need to establish tools to evaluate *in vitro* data and address limitations with its utilization for predicting human health effects. Although *in vitro* data hold great promise, the potential for harm is great – incorrect toxicity predictions could lead to grave failures in protecting public health. Thus, the development of a rigorous framework integrating the best available scientific evidence from *in vitro*, human, and animal evidence for decision makers to base policies and actions on is vital to ensure the protection of public health.

## 3 The use of AOPs for *in vitro* prediction of liver toxicity

Mathieu Vinken gave a lecture on an AOP for *in vitro* prediction of liver toxicity. The liver is a primary target of toxicity induced by xenobiotics. Chemical-induced hepatotoxicity can manifest in several prominent ways, such as cholestasis, steatosis, fibrosis, and cancer, and is a major reason for discontinuation of drug development or withdrawal of drugs from the market. It also is a concern for other sectors, including the cosmetics area (Tabernilla et al., 2021). In this respect, it remains challenging to detect and predict chemical-induced liver toxicity. Indeed, standard animal studies conducted during routine drug development usually pick up about half of all human hepatotoxic compounds, while human-based *in vitro* testing identifies up to 60% of *in vivo* human hepatotoxic drugs (Liu et al., 2011). AOPs are promising tools in that regard, as they may help to predict chemical-induced liver toxicity in a more accurate and mechanistically-anchored way. AOPs are conceptual constructs that portray existing knowledge concerning the linkage between a MIE and an AO through a number of KEs at a biological level of organization relevant to risk assessment. In response to the increasing use of AOPs, the Organization for Economic Cooperation and Development (OECD) together with the US Environmental Protection Agency, the US Army Engineer Research and Development Center, and the European Joint Research Center have introduced the AOP Knowledge Base (AOP-KB). The AOP-KB^1^, is composed of modules, among which the AOP Wiki^2^ provides an open-source interface for rapid, widely accessible, and collaborative sharing of established AOPs, and building of new AOPs. At the time of the workshop, the AOP Wiki contained several AOPs related to chemical-induced hepatotoxicity and liver pathology (Arnesdotter et al., 2021). Some of these AOPs, grouped by three types of hepatotoxicity, were presented, see below. The lecture concluded with an outlook on how to mechanistically assess AOPs using transcriptomics, proteomics, and metabonomics. In addition, a perspective on potential applications of AOP, ranging from chemical grouping to chemical prioritization for assessment for use in integrated approaches to testing and assessment (IATA), was presented.

### 3.1 AOPs for cholestasis (Vinken et al., 2013)

Cholestasis denotes any situation of impaired bile secretion with concomitant accumulation of potentially noxious cholephiles in the liver or in the systemic circulation. Only one AOP on this type of liver toxicity, in particular hepatocellular cholestasis, is currently available in the AOP-KB. The MIE is the direct cis-inhibition of the bile salt export pump. As a result of this, toxic bile salts accumulate in hepatocytes or bile canaliculi. These bile salts trigger a direct deteriorative response and an adaptive response. At the cellular level, the deteriorative response is accompanied by formation of the mitochondrial permeability pore, which leads to mitochondrial impairment, inflammation, the production of reactive oxygen species and, ultimately, to the onset of cell death by both apoptotic and necrotic mechanisms. Because of the latter, cytosolic enzymes, including aminotransferases, start to leak from hepatocytes and cholangiocytes and become measurable in the serum. A hallmark of cholestasis at the cellular level includes the induction of an adaptive response, which is aimed at counteracting bile accumulation and thus cholestatic liver injury. Accordingly, a complex machinery of transcriptionally coordinated mechanisms involving nuclear receptors is activated by bile salts, which collectively decrease the uptake and increase the export of bile salts and bilirubin into and from hepatocytes, respectively. Simultaneously, detoxification of bile salts is enhanced, while their synthesis becomes downregulated. The increased effort of cholestatic hepatocytes to remove bilirubin causes bilirubinuria and hyperbilirubinemia. As a result, a yellowish pigmentation of the skin and the conjunctival membrane over the sclera, known as jaundice, becomes visible. Furthermore, the elevated presence of bile salts in the serum is thought to cause the typical skin itching in cholestasis patients.

### 3.2 AOPs for steatosis (Mellor et al., 2016)

Hepatic steatosis, also called fatty change, fatty degeneration, or adipose degeneration, is the process of abnormal retention of lipids, mainly triglycerides, within hepatocytes. It reflects the impairment of the normal processes of synthesis and elimination of triglycerides. At the time of the workshop, the AOP wiki contained nine AOPs covering hepatic steatosis. Each of these AOPs considers a different MIE, including modulation of nuclear receptors (i.e., aryl hydrocarbon receptor, constitutive androstane receptor, farnesoid X receptor, liver X receptor and pregnane X receptor), suppression of transcription factors (i.e., hepatocyte nuclear factor 4 alpha and nuclear erythroid 2-related factor), activation of serine/threonine kinase 2, and inhibition of peroxisomal fatty acid beta-oxidation. All these MIEs trigger an array of effects, such as enhanced transcription of genes encoding mediators of cholesterol and lipid metabolism, including carbohydrate response element binding protein, sterol response element binding protein 1c, fatty acid synthase and stearoyl-coenzyme A desaturase 1. As a result, *de novo* synthesis of fatty acids is enhanced in the liver. At the same time, fatty acid translocase production is upregulated, which mediates increased hepatic influx of fatty acids from peripheral tissues. Consequently, triglycerides tend to accumulate in hepatocytes. At the organelle level, hepatocellular lipid accumulation may provoke cytoplasm displacement, nucleus distortion, mitochondrial toxicity, and endoplasmic reticulum stress. All together, these effects underlie the acquisition of the typical fatty liver cell phenotype, which in turn causes a clinically relevant increase in liver weight. Hepatic steatosis can develop further to non-alcoholic steatohepatitis, which is characterized by hepatocellular injury and inflammation, and for which an AOP was recently included in the AOP-KB.

### 3.3 AOP for fibrosis (Horvat et al., 2017)

Liver fibrosis is a reversible wound healing response to either acute or chronic cellular injury that reflects a balance between liver repair and scar formation. A central event in liver fibrosis is the activation of hepatic stellate cells, which occurs in 2 phases, namely the initiation stage and the perpetuation stage. In the initiation phase, quiescent hepatic stellate cells become responsive to growth factors. This may be triggered by a variety of signals, including reactive oxygen species and apoptotic bodies originating from dying hepatocytes. In the perpetuation phase, the primed hepatic stellate cells undergo several changes related to proliferation, contractility, fibrogenesis, chemotaxis, extracellular matrix degradation, and retinoid loss, whereby they adopt a myofibroblast-like phenotype. Hepatic stellate cell activation may be counteracted in a resolution phase through apoptosis, senescence, or reversion to the quiescent phenotype. The most progressive form of fibrosis is cirrhosis, which, unlike fibrosis, is considered an irreversible event. At the time of the workshop, the AOP-KB contained one AOP on liver fibrosis in which protein alkylation is considered as the MIE. Different steps at the cellular and tissue level have been defined, including hepatocyte injury and cell death, activation of Kupffer cells, expression of transforming growth factor beta 1, activation of hepatic stellate cells, oxidative stress and chronic inflammation, collagen accumulation, and changes in hepatic extracellular matrix composition.

## 4 Assessing certainty in AOPs (Theme 1)

The main objective of Theme 1, led by Rebecca Morgan and Paul Whaley, was to discuss how certainty in AOPs should be assessed, i.e., how AOPs of higher certainty ought to be distinguished from AOPs of lower certainty. Two angles on this were taken: discussion of the use of the modified Bradford Hill considerations, also known as tailored Bradford Hill criteria, by AOP practitioners in assessing certainty in a putative AOP (Becker et al., 2015; OECD, 2018); and exploring how the use of logic models for analyzing complex interventions in public health systematic reviews might be applied to the AOP context (Rehfuess et al., 2018).

The modified Bradford Hill considerations were chosen for discussion because the GRADE approach for assessing certainty in the evidence is an explicit adaptation of the original Bradford Hill considerations (Schünemann et al., 2011). Given previous discussions of the potential for applying the GRADE approach to assessing certainty in AOPs, it made sense to explore how the Bradford Hill considerations are being used by AOP practitioners. This would help determine whether there are any aspects of their application that are not already addressed within the domains of the GRADE evidence assessment.

The use of logic models in systematic reviews was chosen for discussion to determine what lessons there might be in the assessment of complex interventions for assessing certainty in AOPs. Since AOPs are simplifications of complex causal networks, but in a biological setting, it was considered that logic models for complex interventions may be informative in applying systematic methods from health research to the AOP context.

### 4.1 Modified Bradford Hill considerations in AOP/MoA analysis

Bette Meek introduced the use of modified Bradford Hill considerations in AOP and MoA analysis. Descriptions of MoAs and AOPs facilitate systematic integration and assessment of mechanistic data in hazard assessment from a broad range of sources. Formalized description and analysis of the extent of supporting evidence for these pathway descriptions supports their use for various applications in testing and assessment.

Selected Bradford Hill considerations form the basis for assessment of the extent of supporting evidence in formalized descriptions of AOPs. These considerations, modified somewhat from their initial characterization to assess causality in epidemiological studies and adopted in international frameworks in MoA analysis include biological plausibility, essentiality, and empirical support.

The considerations, defined to address aspects critical in regulatory acceptance, are also rank ordered to reflect their relative importance in assessing the extent of supporting mechanistic data. Examples of the types of datasets associated with high, moderate, and low confidence accompany each of the modified considerations in program guidance (OECD, 2018). These designations are based on the extent to which evidence supports expected patterns across levels of biological organization and data sources, including studies in humans, animals, and *in vitro* or *in silico*. For AOPs in the AOP-wiki, the extent of confidence for these considerations in the KE, KER, and the AOP overall are also illustrated in a user-friendly summary graphical interface to guide appropriate application.

The modified Bradford Hill considerations are “expert informed” and application driven, reflecting experience in regulatory application of MoA analysis and evolution in the OECD AOP development program through collaborative interface of the research and regulatory communities. They draw on accumulated experience to contribute to the delineation of the content of reporting templates for individual KE, KER, and the AOP overall.

In addition to assessing confidence for each of the considerations as high, moderate, or low, developers provide text rationales to justify their designations based on the provided examples for components and the overall AOP. The OECD Extended Advisory Group on Molecular Screening and Toxicogenomics reviews WoE descriptions internally for compliance with program guidance. Descriptions are reviewed subsequently by a team of external scientific subject experts and approved by the OECD Working Group of the National Coordinators of the Test Guidelines Programme and the OECD Working Party on Hazard Assessment.

Specific definition of the modified Bradford Hill considerations and provision of examples of the nature of supporting datasets giving rise to different levels of confidence and associated templates for description of the relevant features of the supporting datasets contributes to the consistency and transparency of AOP descriptions. Additionally, they increase common understanding among the research and regulatory communities of the elements and types of data or studies, contributing to confidence in AOPs/MoA for regulatory application.

For hazard characterization, the focus in WoE assessment for AOPs/MoA on expected patterns across different levels of biological organization supported by different types of data from a range of sources addresses the synthesis or integration step of data assimilation and assessment. As such, it is one of a limited range of methods available to address integration across lines of evidence, arguably the stage most influential for application.

The approach follows a number of previous requisite steps in considering data as summarized in Box 1, including definition of the relevant question and systematic identification, organization, and selection of relevant and reliable studies (see for example, Rhomberg et al., 2013; Martin et al., 2018). These integrating characteristics also distinguish the consideration of mechanistic evidence based on the modified Bradford Hill considerations for AOPs/MoA from lower-level constructs such as the key characteristics of carcinogens (Smith et al., 2016), which identify potential KE without organization or discrimination relative to regulatory application (Tab. 1).

Since the modified Bradford Hill considerations address critical considerations in interpretation of mechanistic data for regulatory application, WoE analysis for MoA/AOPs informs focus for efficient investment of resources to ensure transparency and defensibility in critical areas. This could include, for example, systematic reviews for specific questions, taking into account the specific nature of AOPs and MoA, which address how chemicals induce effects across levels of biological organization rather than associating cause and effect at one level only (i.e., hazard identification).

As hypothesis-based integrating constructs based on prior biological knowledge, AOP/MoA development requires specialized, iterative literature search strategies. Early consideration in problem formulation of WoE considerations for integrating mechanistic constructs such as AOP/MoA is helpful, then, in identifying relevant patterns across studies and lines of evidence based on considerations relevant to regulatory priorities. It also facilitates the early integration of hazard and mechanistic data, considering patterns of relevant determinants across different levels of biological organization such as empirical support.

### 4.2 Logic models for assessing complex interventions

Elizabeth Kristjanson introduced the application of logic models in systematic reviews. Logic models, also referred to as conceptual models, analytical models, concept maps, and theory of change, are usually graphical explanations of intervention and the mechanisms through which it has impacts. They have been applied in the context of systematic reviews since the early 2000s, where they are developed in the early stages of protocol development. They offer a solution to explicitly capture the steadily increasing complexity of systematic reviews (Anderson et al., 2011), especially for reviews addressing public health or nutrition questions, and support various review steps, from framing of the question and scoping the review to planning the analyses. There are three different types of logic models, which are distinguished by how they have been developed (Rehfuess et al., 2018):

- *A priori*, which are developed as close as possible to the onset of the systematic review, and which are not changed during the review
- Staged, which specify points at which new information from the review is expected to result in changes to the model
- Iterative, which can be modified at any time during the review, accounting, for example, for new learnings or societal changes

These types primarily differ in how they balance transparency and flexibility.

### 4.3 Report on Theme 1

There were three principal lessons from the break-out group discussions, which are discussed here in detail.

#### 4.3.1 The importance of the differences between research and regulatory contexts when assessing certainty in biological knowledge

While the structured thinking being applied in AOPs is of high potential value for developing methods for assessing indirectness of evidence in systematic reviews, the differences between regulatory and research contexts should not be underestimated when adapting AOP approaches to the systematic review context.

The development of AOPs is driven by the need to formalize biological knowledge in such a way that it can inform regulatory decisions. The fact that AOP development is an initiative of the OECD is a demonstration of its intent for regulatory use (OECD, 2018). It is important, therefore, that an endorsed AOP should not be conflated with a full description of the biology underpinning an exposure-outcome relationship. The AOP is a formalization of the most commonly measured elements of the relevant biology, developed for the purpose of helping make decisions about which NAM could be used in standardized regulatory toxicology test strategies, and for organizing mechanistic data in specific chemical assessments. Nor should it be assumed that the characteristics of the evidence that are assessed because of regulatory priorities will map onto those that systematic reviewers would necessarily consider when summarizing what the evidence is saying in answer to a research question. There may be some overlap between the two contexts, but they are not the same.

The intent of AOPs is to organize elements of toxicity-related biology that predict apical outcomes to a degree that assays based on those elements may provide evidence of whether a chemical presents unacceptable health risks of a sufficient level of certainty for use in regulatory contexts. The function of the modified Bradford Hill considerations is to facilitate the assessment of whether a biological event, or a relationship between two events, is sufficiently certain that it can be described as a KER, and as such can be used in regulatory decision-making. The considerations also derive from past regulatory experience in MoA analysis. The context for application of the modified Bradford Hill considerations in developing an AOP is therefore quite different to assessing the certainty of a biological mechanism in a systematic review. Hartung et al. (2013) stressed earlier the key role of the Bradford Hill criteria for mechanistic validation of AOP.

That said, relating biological knowledge to an AOP can be viewed as a process amenable to systematic methods, on the condition that it is understood as a move from observed associations (as can be discovered by, e.g., mining the literature) to identifying those relationships in which we have enough certainty that they can be used to reliably predict health outcomes. The higher the certainty in a relationship between a biological event and a final outcome, the less the evidence would need to be rated down when using that event as an indirect endpoint in a systematic review. Whether the event is sufficiently predictive for regulatory use can be treated as a separate question with its own supplementary certainty criteria or by setting the bar for certainty in the relationship at the level that decision-makers determine to be appropriate.

#### 4.3.2 The broad applicability of logic models in assessing certainty in biological knowledge, with major challenges in data volume

Logic models are conceptual constructs that model complex causal relationships between risk factors and health outcomes (Anderson et al., 2011). They are often presented as directed acyclic graphs (DAGs). Because there is no fundamental difference between exogenous and endogenous influences on health outcomes, biological influences on health outcomes can also be presented as DAGs – indeed, under certain constraints (e.g., lack of loops among KE), AOPs can be viewed as a type of DAG. While biological mechanisms are amenable to representation as logic models, and this is indeed a fundamental insight of the AOP approach, the volume of evidence that needs to be handled in a logic network in a biological setting is vast and may be impractical to address with current manual SR approaches.

As discussed at a previous workshop (De Vries et al., 2021), GRADE might be applied to logic models as well as exposure-outcome pairs; at this workshop, we revisited the idea with a different group consisting of a larger number of toxicologists to determine if there are additional issues that need to be considered.

**Fig. 1:**
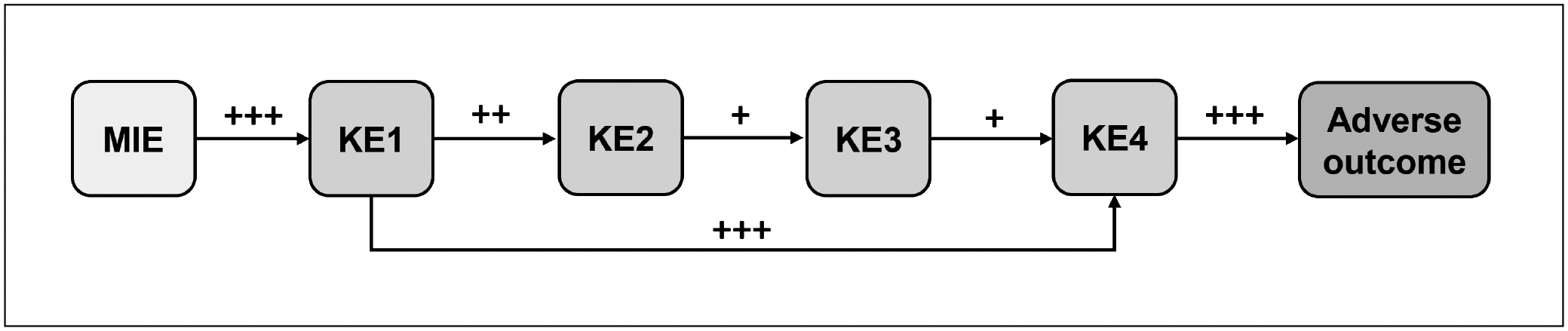
Certainty in a given path of a network depends on the certainty in each individual event-event relationship that constitutes the network The higher the certainty in a relationship, the more predictive (and less indirect) the upstream event is as a surrogate for a downstream event (MIE, molecular initiating event; KE, key event).

The group concluded that the principle of applying GRADE to biological networks is, in theory, relatively straightforward: each event-event relationship in the network can be treated as if it were an exposure-outcome relationship; evidence relevant to assessing the causality of the relationship can therefore be systematically reviewed and assessed for certainty. If this is done for each event-event relationship in a biological network, then an understanding of the certainty of the overall network will emerge (see Fig. 1). Certainty in a given path of a network depends on the certainty in each individual event-event relationship that constitutes that path. How to integrate those uncertainties to obtain the certainty for the path is not clear and is a matter for further research.

The group also concluded that one of the major challenges in applying systematic review methods to the context of AOP development is the volume of evidence being assessed. AOPs are also integrating constructs, where patterns across different levels of biological organization are highly influential in determining certainty in the biological network. Aspects of certainty for the elements are considered in the context of their contribution to an integrating construct across different levels of biological organization – hence, the modified Bradford Hill considerations. Handling such a large volume of evidence, consisting of multiple interdependent elements, in a systematic manner is a considerable challenge and may require rapid review methods or that evidence assessment be significantly supported by automated approaches.

#### 4.3.3 Differences in concepts, vocabulary, and reasoning processes

A major challenge in applying systematic review methods to AOP development is in the different vocabulary and reasoning processes employed in the two approaches; considerable work is required to interpret and translate processes and vocabulary if the lessons from one are to be fully applied to the other. This is to be expected, as the two approaches have developed independently in different disciplinary and regulatory contexts. A particular issue is that it is not immediately apparent how the drivers of certainty in defining AOPs relate to drivers of certainty when applying GRADE in systematic review. While it is relatively easy to conceptualize how a GRADE assessment of individual KER might be conducted for a putative biological network, the same does not hold for the development of a complete AOP. This is because AOPs utilize different ways of operationalizing the same process (i.e., arrive at certainty via different means) and introduce additional concepts that may need to be accommodated in the GRADE approach. That SR methodologists and developers of AOPs come from different communities with, at this stage, only limited shared experience, makes the task of shaping SR methods for AOP development (and *vice versa*) very challenging (see Tab. 1). Further meetings and the ongoing work of multi-stakeholder networks such as the EBTC would seem to be of particular value for this purpose.

## 5 Literature-based AOP development (Theme 2)

The main objective of Theme 2, led by Michelle Angrish and Steve Edwards, was to consider whether systematic review techniques traditionally used to assess human and environmental effects of chemical exposure could be adapted to the development of AOPs. This would allow risk assessors to leverage the power of the AOP for integrating toxicological data without sacrificing the rigor and transparency provided by SR. As a basis for discussion, the formal process for developing AOP and a case study of thyroid hormone pathway disruption in humans for applying SR methodology to AOP development were chosen as topics for the theme-specific presentations.

### 5.1 Formal process for developing AOPs

Steve Edwards presented the formal process for developing AOPs. AOPs connect early perturbations of a biological system, which can be measured via *in vitro* methods, to adverse outcomes that impact humans (or wildlife) populations (Ankley et al., 2010). The AOP framework is specifically structured to achieve this purpose and has two primary components: KE and KER (Villeneuve et al., 2014). The KE are focused on what we measure, so they will drive assay development, promote biomarker identification and evaluation, and provide a framework to integrate these mechanistic measurements with apical endpoints such as cancer in humans. Because of this, the emphasis when defining a KE is on the measurability of that event.

Ideally, a KE would include measurements that can be routinely used to evaluate whether a chemical is likely to operate via a given AOP. However, in most cases there are KE within the AOP that are easily measured and others that are measurable with a higher degree of difficulty. This is also acceptable because the latter KE are not likely to be needed once the AOP has been confidently established because routine monitoring of chemicals can be done through the more easily measured KE. At some point, most, if not all, routine measurements will eventually move to *in vitro* systems, which are not only cheap and easy to measure but also eliminate the need for laboratory animals. *In vivo* measurement of later KE can be reserved for chemicals where the uncertainty associated with *in vitro* predictions is unacceptable.

In order to reduce the number of KE measurements required for monitoring activation of an AOP, the user of the AOP must have confidence that early KE are necessary for the occurrence of the later KE within the context of a given AOP (Becker et al., 2015). The second component of the AOP directly addresses this need by clearly defining the relationship between each pair of KE and summarizing the evidence supporting the causal relationship between the events (Villeneuve et al., 2014). In cases where strong evidence supports this causal relationship, an IATA would only need to incorporate assays monitoring one KE as the other would logically follow from activation of the first. Should evidence supporting all KER be strong, an argument could be made for the measurement of the MIE alone.

The decision to focus on early KE depends on several aspects. First, evaluations of the assays in addition to the confidence in our understanding of the toxicological mechanism provided by the AOP and the confidence in the assays used to inform the KE and KER has to be considered. A suite of *in vitro* assays covering the early KE in the AOP should be a practical means for monitoring activity through that AOP if the confidence connecting all downstream KE is high.

Next, the level of quantitative understanding of the relationships between each pair of KE should be considered (Perkins et al., 2019). In cases where there is high confidence in the KER but little quantitative understanding of those relationships, assays for early KE can be used to identify potential hazard but not to assess the risk from a chemical. Getting a fully quantitative AOP is costly and time consuming, but even qualitative AOPs can assist with risk predictions. A great example is in support of read-across applications. A chemical could be screened in low-cost, high-throughput assays to provide increased support for the structural and mechanistic class of chemicals to be used for the read-across prediction of potential risk.

Finally, the domain of applicability of an AOP needs to be taken into account. Since AOPs are chemically agnostic (Villeneuve et al., 2014), this is distinct from the use of domain of applicability in the context of cheminformatics and QSAR. However, the intent of the phrase is the same. For AOPs, three domains of applicability are considered: taxonomic (i.e., species), sex, and life stage. These can be specified for individual KE, relationships, or for the AOP in general. The convention in how the three are defined is slightly different. For taxonomic applicability, the species in which the AOP, KE, or relationship was explicitly observed is generally recorded. For other species where the AOP is likely applicable, the decision is left to the user. For sex and life stage, these are generally specified when the AOP is specific to a certain sex or life stage. In cases where the AOP is relevant in males and females, or across all life stages, these annotations are generally left blank with the assumption of general applicability.

With all of these considerations in mind, the OECD Extended Advisory Group on Molecular Screening and Toxicogenomics, which oversees the OECD AOP Development Programme, has published a handbook to assist AOP authors in correctly defining and evaluating AOPs (OECD, 2018). This document provides detailed guidance on creating and evaluating an AOP coupled with links to instructions for entering the information into the AOP-Wiki, which is the component of the AOP-KB focused on defining and evaluating AOPs. As AOP applications continue to grow (Ede et al., 2020; Knapen et al., 2020; Wittwehr et al., 2021), the need for consistency and accuracy in defining and evaluating AOPs is more important than ever. It must be noted, however, that AOPs do not need to be exhaustively annotated to support environmental and public health decisions. As long as the AOP is developed via a transparent and open process, even AOPs with less documentation and/or lower confidence may be applicable for prioritization of chemicals for screening and other similar uses.

### 5.2 Case studies for applying SR methodology to AOP development

The second theme-specific talk was given by Michelle Angrish, who presented a case study for applying SR methodology to AOP development. SR methods are a natural fit for AOP development, providing a mechanism for meeting the expectation that an AOP will include the most relevant and robust information. Even though practical application of SR methodology is labor-intensive and costly, there are aspects of SR workflows (e.g., foundational methods and concepts, open-source, and freely accessible tools, etc.) that offer flexibility under the rubric of current AOP development guidelines. These advantages include leveraging pathway-based systematic methods that add transparency, traceability, ease in updating, and reduced bias in selecting data and/or information mapped to AOP concepts.

Therefore, the initial goal of this case study was to scope out the feasibility of a NAM that incorporates aspects of systematic methods (namely evidence mapping) guided by AOP concepts to prioritize and screen chemicals based on potential thyroid hormone pathway disruption in humans. Given that expert knowledge has identified several possible sites of chemical-molecular interaction as well as neurological and developmental outcomes related to thyroid pathway disruption (Gilbert et al., 2012), a pilot evidence mapping project was conducted. Reviewers were tasked with screening, at the title and abstract level, and tagging studies for the MIE sodium/iodide symporter (NIS), other MIEs, evidence type, chemicals, assay target/type, and other mechanistic information. While the purpose of the pilot was to design the overall systematic method, the project quickly ground to a halt, mostly due to variation in language. Study methods and findings are almost exclusively recorded using highly variable natural language. This presents a significant semantic challenge because linguistic variation can obscure concepts and relationships needed for information retrieval, interpretation, and contextualization (i.e., thyroid hormone vs. thyroid gland vs. thyroid disease). This natural variation led to errors in screening decisions that negatively affected the validity of the reviews.

Acknowledging the desire to balance the advantages that could be gained using systematic methods with the downside of natural language variation, the pilot was retooled to leverage the AOP framework (Ankley et al., 2010). Currently, AOP information is captured in a schema that maximizes the benefits established by the findable, accessible, interoperable, and reusable (FAIR) data principles by applying ontology-based annotations to concepts within the biologically structured AOP framework (Ives et al., 2017). Therefore, the pilot shifted toward a step-wise goal of literature inventory information digitization that would feed back on the screening process to improve screening decisions. This stepwise approach meant that information captured in an evidence map (environmental science vocabulary) requires transformation into controlled vocabularies (unambiguous and non-redundant) that can be mapped to ontologies amenable to the AOP framework.

Realizing that systematic review methodology is conducted in a step-wise structured manner that is amenable to technological innovation, strategies to address several advances and lingering challenges faced during application of computationally intelligent approaches are being explored.

### 5.3 Report on Theme 2

A challenge in applying systematic review methods in AOP development is considering the depth of coverage. A single AOP is a broad topic that may include multiple hypotheses that could each be an independent SR. While SRs are well-suited to hypothesis-driven and focused questions that are confirmatory, it was recognized that SR could quickly become unwieldly in AOP development. Systematic evidence maps rely on the same principles as SR but are better suited to exploration (Wolffe et al., 2019). It is anticipated that AOP development will include a combination of a broad survey of the literature related to an AOP (i.e., evidence mapping) followed by a more detailed evaluation of the evidence supporting the connection between pairs of KE within the AOP (i.e., SR for KER).

For example, evidence maps provide the same level of consistency, transparency, and documentation as an SR but present summaries of the evidence. Such summaries are useful for refining an AOP developer’s approach based on the available evidence and goals of AOP development. An additional challenge is developing the search strategy for finding data that will inform the evidence map, understanding that the broader the search, the more effort is required to screen the retrieved results. In the context of AOP, the initial literature search strategy may consider the intended application of final AOP, resources as well as time, and tailor the screening criteria accordingly. For example, an AOP developer may start with a broad search strategy and then refine the search results based on the available information or identify additional strategies needed to cover gaps in the evidence map. Search results may be refined in terms of taxonomy, MIE, KE, AO, test method, etc.

To bound the search space, AOPs are defined as connecting a single MIE to a single adverse outcome (AO). In cases where the search is initiated from the MIE or AO, a decision must be made as the search is expanded regarding which MIE or AO will serve as the second anchor. For selecting an AO, the prevalence and severity of the disease are important factors. For selecting an MIE, the number of chemicals and relative toxicity of chemicals in the class would be factors for consideration. In cases where the starting point is an intermediate KE, a choice can be made to create a partial AOP with that KE as an anchor or to identify both an MIE and an AO as the search is expanded. All of these decisions should be driven by the planned use of the resulting AOP. If multiple interacting AOPs are desired, the overlapping KEs from the first defined AOP can be used as the initial search for the second and so on.

**Fig. 2:**
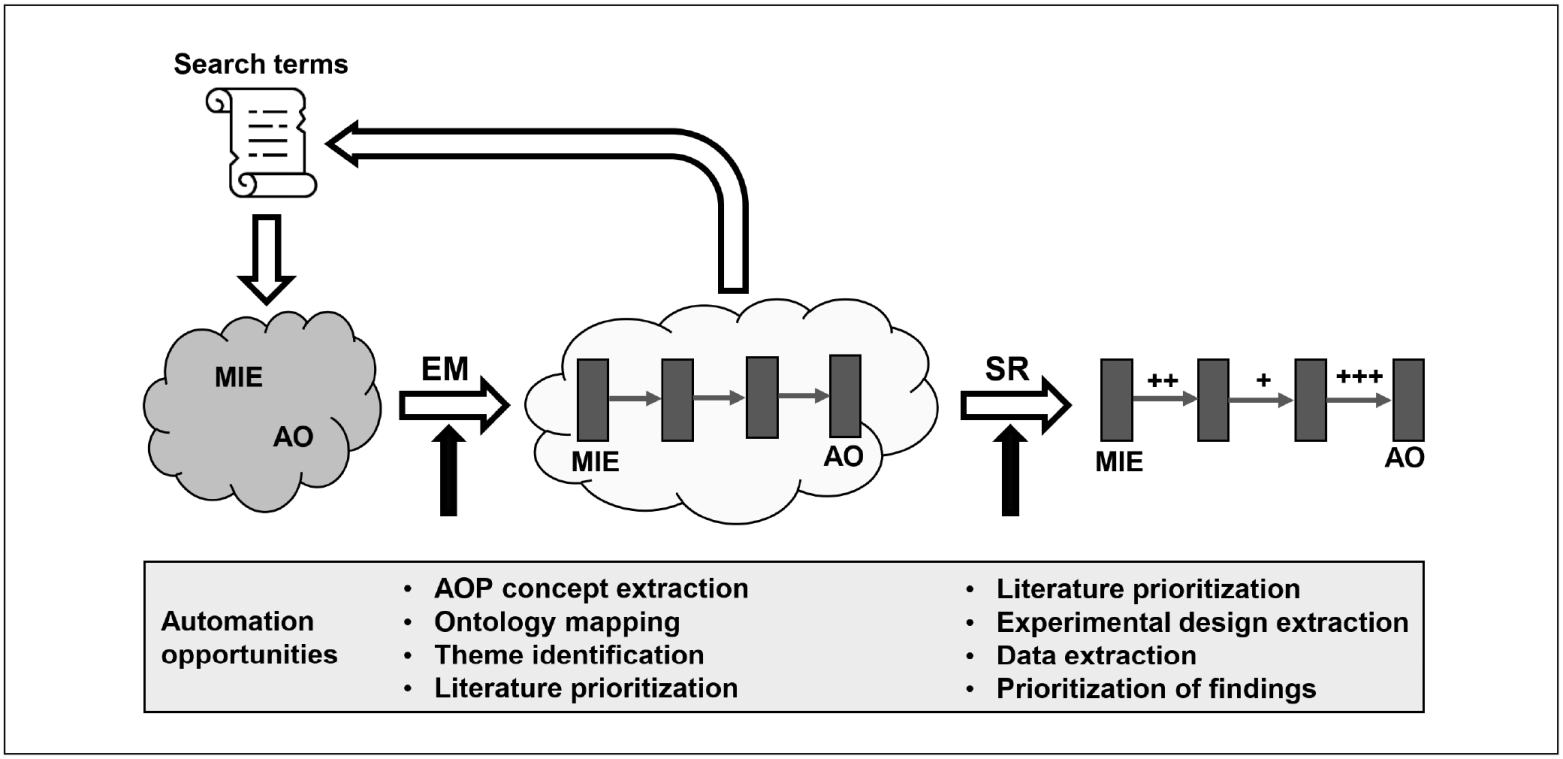
Systematic review-based AOP workflow EM, evidence map; SR, systematic review; MIE, molecular initiating event; AO, adverse outcome; +/++/+++, certainty rating in key event relationships

Given the high correspondence between evidence mapping and the current processes used to define the overall structure of an AOP, the AOP development workflow shown in Figure 2 is recommended. As described above, an initial set of search terms focused on the disease, assay, or chemical class of interest is used to collect the initial literature corpus. Evidence map approaches are then used to identify potential KEs and KERs, which will allow the expansion of the search space to include terms that define the identified KEs. This process is repeated until one or more AOPs are identified that include an AO and preferably include the MIE. These putative AOPs can then be further refined via SR.

Once the KEs and their connectivity within an AOP have been defined, the next step in the AOP development process is evidence evaluation. Currently, three main elements are considered when evaluating the evidence in support of an AOP (OECD, 2018). The first two are evaluated for each pair of KEs. The biological plausibility or extent of knowledge of the mechanistic relationship of each KE pairing is the primary factor determining the confidence in that KER. In some cases, however, the biological mechanisms connecting two KEs have been studied for decades, the causal pathway has been worked out in detail via a series of seminal studies, and the knowledge is considered dogma. The second factor considered when evaluating the relationship between two KEs is the empirical support for a causal relationship between them. This includes temporal concordance where activity for the upstream KE precedes that of the downstream KE. In cases where chemical perturbations are examined, there is also dose concordance where the upstream KE is seen at lower doses than the downstream KE. The final consideration when evaluating an AOP is the essentiality of the KEs. While this is recorded for the AOP as a whole (i.e., a KE has been shown to be essential for the AOP to occur), the experimental data that informs this decision is captured during the evidence evaluation of the KERs (e.g., knocking out an upstream KE to evaluate the causal relationship for the pair also informs the essentiality of the KE for the AOP).

SR approaches can improve the transparency, consistency, and accuracy of AOP development and include approaches for WoE evaluation and overall assessment of the AOP (Fig. 2). The AOP Developer’s Guidance document does include guidance on assessing the WoE and overall assessment of the AOP (OECD, 2018), however, the evaluation criteria were not developed considering the processes currently implemented in SR. It may be possible to follow a process similar to the GRADE categories for the strength of a recommendation in deciding whether an AOP is suitable for the proposed use, however WoE considerations were not the specific focus of Theme 2. Regardless, while regulatory entities seeking to use AOPs in regulatory purposes will likely apply organization-specific criteria for WoE considerations, the community and AOP-related efforts in general would benefit from AOP development processes that are clear, transparent, and documented.

While challenges remain with respect to applying systematic methodologies to AOP development, this goal is achievable and should be pursued. The AOP framework provides a structured way of representing mechanistic data that makes it straightforward for decision makers and stakeholders to review the evidence in support of a public health decision. The use of systematic methods to populate this framework will result in accuracy, consistency, and transparency in the collection and interpretation of the data underlying each AOP. In addition, advances in artificial intelligence (AI) such as natural language processing and machine learning may add efficiency to the process (Marshall and Wallace, 2019; O’Connor et al., 2020; Bozada et al., 2021). These AI tools are already integral to SR workflows and include machine assisted topic clustering and prioritization of search results, machine assistance for study screening, normalization of terminology using controlled vocabularies and ontologies, etc., with the advantage of a human-in-the-loop model. In the future, it is anticipated that automation of key steps in the workflow will reduce the overall time required and increase the throughput of AOP development without sacrificing the advantages of the systematic approaches.

## 6 Integrating certainty in AOPs and non-animal evidence into decision frameworks

Theme 3, led by Ingrid Druwe and Sebastian Hoffmann, addressed the topic of how AOPs can inform the development of NAM, especially non-animal experimental studies. For this workshop, the topic was broadened to discuss how certainty in AOPs (see Theme 1) and in the evidence derived from NAMs used to inform an AOP interact and how this process can be systematically considered in frameworks used to derive chemical risk assessment conclusions and decisions.

The first theme-specific presentation aimed at demonstrating commonalities and differences between chemical risk assessment frameworks to illustrate the extent of applicability of the topic to various contexts. It stressed that NAMs will increasingly provide more of the evidence to inform future toxicology paradigms, regardless of the framework. The second presentation introduced the GRADE approach for synthesizing evidence and developing recommendations in order to explore potential similarities with chemical risk assessment frameworks.

### 6.1 Commonalities and differences of chemical assessment frameworks

Ingrid L. Druwe and Sebastian Hoffmann presented commonalities and differences of chemical assessment frameworks. Various chemical assessment frameworks exist, which aid regulators and risk assessors to fulfil their mandates, particularly to support decision-making related to human health hazard identification and risk assessment. These frameworks serve multiple and related purposes such as: hazard assessment, classification, and labeling, risk assessment, prioritization, restriction, authorization, and support of other legal actions. They use relevant information of diverse origins, such as epidemiological, animal (*in vivo*), and NAM, e.g., *in vitro* and computational approaches. While in most cases animal evidence is used to inform assessments, ethical and, more recently, political considerations have started to induce a shift towards NAM evidence.

At US EPA’s Center for Public Health and Environmental Assessment (CPHEA), the choice of framework(s) is determined based on the purpose of the assessment and the amount of information available to inform a chemical assessment. For example, Peer Provisional Reference Toxicity Value (PPRTV) assessments have limited data and are used to aid the Office of Land and Emergency Management (OLEM), while Integrated Risk Information System (IRIS) toxicological assessments, which identify and characterize health hazards of environmental chemicals, are data-rich and are used by a variety of stakeholders. PPRTVs integrate human-relevant information and use expert-driven read-across methodologies to fill toxicokinetic and toxicodynamic data gaps to derive a health risk value for a chemical of interest. In PPRTVs, a chemical framework such as the AOP framework may be used to anchor information from a given assay to a KE that leads to an AO of interest to regulators. In contrast, for IRIS toxicological assessments, a wealth of evidence from various origins is available, which is assessed and integrated to identify human health hazards and derive risk values. In the drafting of IRIS assessments, AOP frameworks are useful in organizing and visualizing the WoE in support of a given AO. The OECD has structured regulatory conclusion processes using diverse types of evidence by IATA. The generic IATA framework comprises three parts: 1) problem formulation and gathering of existing information, 2) WoE assessment and, if 2) is inconclusive, 3) iterative generation of additional information and WoE assessment, which is repeated until the information is adequate to draw a conclusion (OECD, 2016; Casati, 2018), as shown in Figure 2. While the first IATA proposed by the OECD originates from the pre-AOP era, the usefulness of AOP to develop IATA has been described more recently (OECD, 2016). One of the first AOP-based IATA has been implemented in the European chemical regulation REACH to obtain skin sensitization classifications (Pistollato et al., 2021).

In summary, there are numerous commonalities of chemical frameworks, including structural problem formulation, and collecting and assessing of evidence from diverse data streams. As more mechanistic and NAM evidence becomes available, these chemical frameworks will continue to evolve to make best use of this evidence in decision-making. Differences in the use of chemical frameworks include methodological uses, e.g., uncertainty assessments, evidence integration, and legal requirements, as well as retrospective versus prospective elements.

### 6.2 Relation of the evidence-to-decision (EtD) framework to chemical assessment frameworks

Elena Parmelli and Andrew Rooney’s presentation was focused on the relation of the EtD framework to chemical assessment frameworks to explore potential similarities.

Andrew Rooney explained how certainty is assessed in evidence evaluations of the National Institute of Environmental Health Sciences (NIEHS) Division of the National Toxicology Program (DNTP) Integrative Health Assessments Branch (IHAB), which serves as an environmental health resource for public and regulatory agencies (Rooney et al., 2014). OHAT conducts literature-based evaluations to assess the evidence that environmental substances cause adverse health effects using and developing systematic review methods. Targeting all relevant data types (human, animal and mechanistic/NAM), a systematic review is carefully planned, and the process is outlined in a protocol. All relevant studies identified are further processed through data extraction, risk-of-bias assessment, and data analysis. Subsequently, the certainty in the body of evidence that findings from a group of studies reflect the true relationship between exposure to a substance and effect is rated for each data type/evidence stream. Similar to approaches by the GRADE Working Group, OHAT has defined initial confidence ratings for the various data types based on study features such as level of exposure control that roughly corresponds to study designs. For example, experimental animal studies are initially rated to be of high confidence, and cross-sectional human studies are initially rated as low (on a scale of very low, low, moderate, high).

Bodies of evidence are then assessed for factors that can potentially increase confidence, such as effect size and dose-response, and factors that can potentially decrease confidence, such as risk-of-bias and indirectness. The final confidence is then determined by applying these assessment results to the initial confidence. Ultimately, the evidence, primarily human and animal studies, is integrated using a predefined approach based on the final confidence levels to obtain a conclusion, which is associated with an indicator (as not classifiable, suspected, presumed or known).

Elena Parmelli continued the presentation by introducing the GRADE EtD framework, which was developed within the 5-year project DECIDE^3^ (Developing and Evaluating Communication strategies to support Informed Decision and practice based on Evidence), which was co-funded by the European Commission (Alonso-Coello et al., 2016a,b). The objective of the project was to improve the dissemination of evidence-based recommendations by building on the work of the GRADE Working Group to develop and evaluate methods that address the targeted dissemination of guidelines.

The main purpose of the GRADE EtD framework is to structure evidence in a clear and transparent way to inform recommendations and decisions in different contexts (Parmelli et al., 2017; Li et al., 2018; Moberg et al., 2018). It is structured in three main sections that reflect the main steps to go from evidence to a decision: formulating the question, summarizing and assessing the evidence, and drawing conclusions. To facilitate its use, the EtD framework has been implemented in the software GRADEPro^4^.

In the GRADE EtD, the criteria that are used to assess interventions or options, the judgments made by the panel for each criterion, and the research evidence and additional considerations used to inform each judgment are made transparent and explicit. The criteria of the GRADE EtD framework are listed in Table 2. Using it helps consider each important factor that determines a decision (criteria) providing a concise summary of the best available research evidence to inform judgements about the pros and cons of each option (intervention). This forms the basis for a structured discussion and a transparent decision.

The GRADE EtD framework provides a structured and transparent approach to support decision-making informed by the best available research evidence, while making the basis for decisions accessible to different stakeholders. It can also be used to facilitate dissemination of evidence and decisions, enabling their adoption/adaptation to different contexts. It is, for example, used in the European Commission Initiative on Breast Cancer to develop the recommendations for the European breast cancer guidelines on screening and diagnosis^5^.

### 6.3 Report on Theme 3

**Fig. 3:**
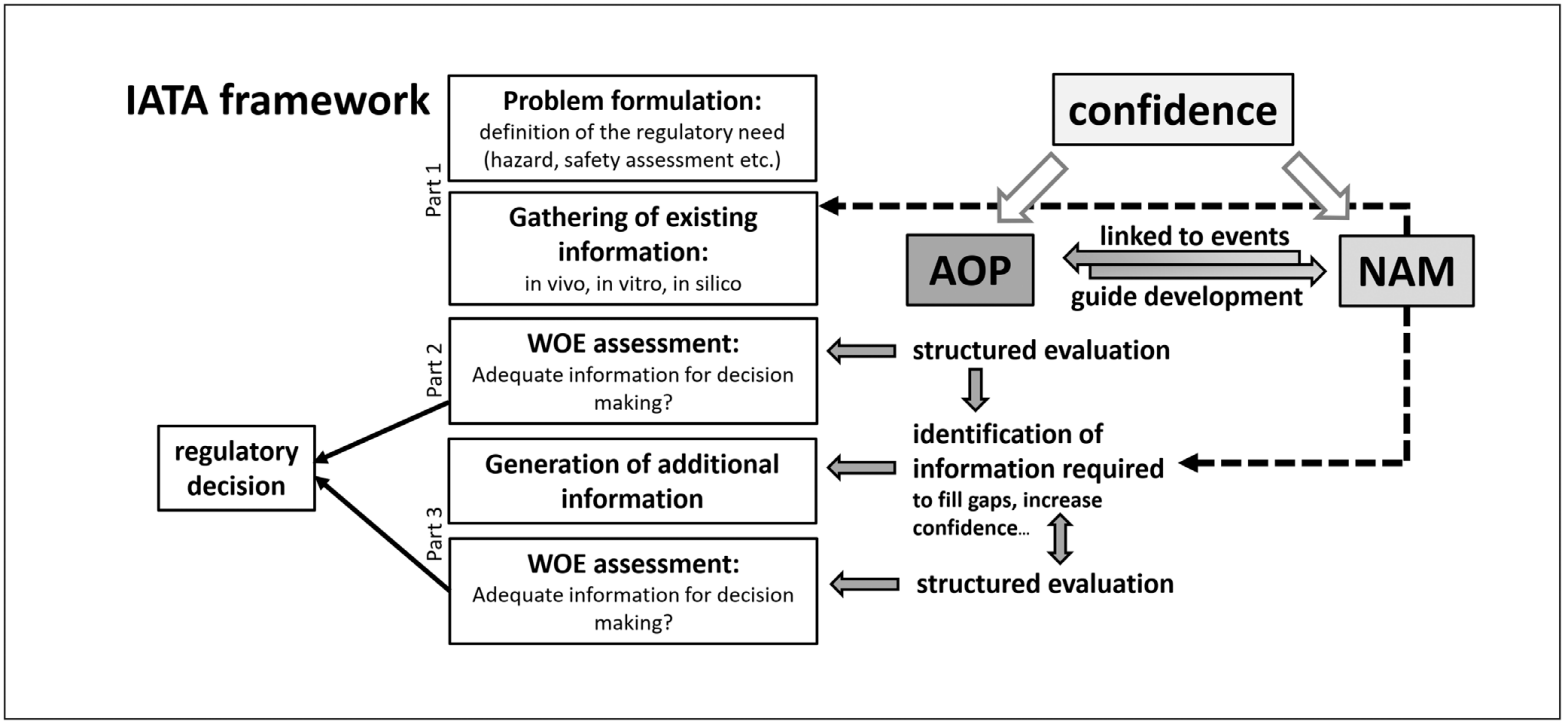
Scheme placing AOP and NAM in chemical risk assessment frameworks based on Figure 4 of the OECD “Guidance Document for the Use of Adverse Outcome Pathways in Developing Integrated Approaches to Testing and Assessment (IATA)” (OECD, 2016)

The scheme of Figure 3, which places NAM, AOP, and the associated certainty considerations into a chemical risk assessment framework (here an IATA was used as an example), was used to visualize the context and to anchor the discussion of Theme 3.

A fundamental issue that came up continuously throughout the discussion was the need for a clearly defined terminology. This was particularly important for the use of “certainty” and “uncertainty”, which are commonly used in the context of chemical risk assessment, and “confidence”, which is used by GRADE, and how trust and trust-building is related to them. Also, the term “quantification of AOP” was used in two contexts: a) as a means to increase confidence in KER and b) as a way to provide points of departure (POD) useful in regulatory contexts. It was concluded that a glossary would be helpful to build a common understanding, e.g., by expanding the glossary of Hoffmann et al. (2017).

Another fundamental need to successfully implement NAM evidence and AOP in chemical risk assessment is stakeholder trust. This trust can only be built over time. The methodological rigor and transparency inherent to evidence-based and systematic approaches can contribute to trust-building and can help to objectively assess current approaches, e.g., in terms of reproducibility, as a point of reference. In this context, the potential of AOP, NAM, and IATA to provide mechanistic information required for population-specific assessments, especially when addressing vulnerable populations, which is hardly possible with current approaches, was stressed (Dornbos and LaPres, 2018; Burnett et al., 2019; Darney et al., 2021).

It was further discussed that the gradual change to NAM and AOP-based chemical risk assessment would also require a fundamental change in chemical risk assessment methodology with its current focus on POD. If the concept of a POD were maintained, it would be a major challenge to obtain POD from integrating evidence from multiple NAM sources. While a NAM-based risk assessment framework with a POD appears to be applicable to some human health endpoints (Gilmour et al., 2020), this may not be the case for other, more complex endpoints. Moreover, frameworks need to shift the focus from adverse outcomes assessed via animal experiments to AOP-structured information on pathway perturbations potentially providing human relevant evidence more directly. This approach also offers to investigate broader questions, going beyond the traditional focus of current chemical risk assessment as demonstrated by an investigation of the potential role of plant protection products in human Parkinson’s disease and childhood leukemia (EFSA PPR Panel, 2017).

**Fig. 4:**
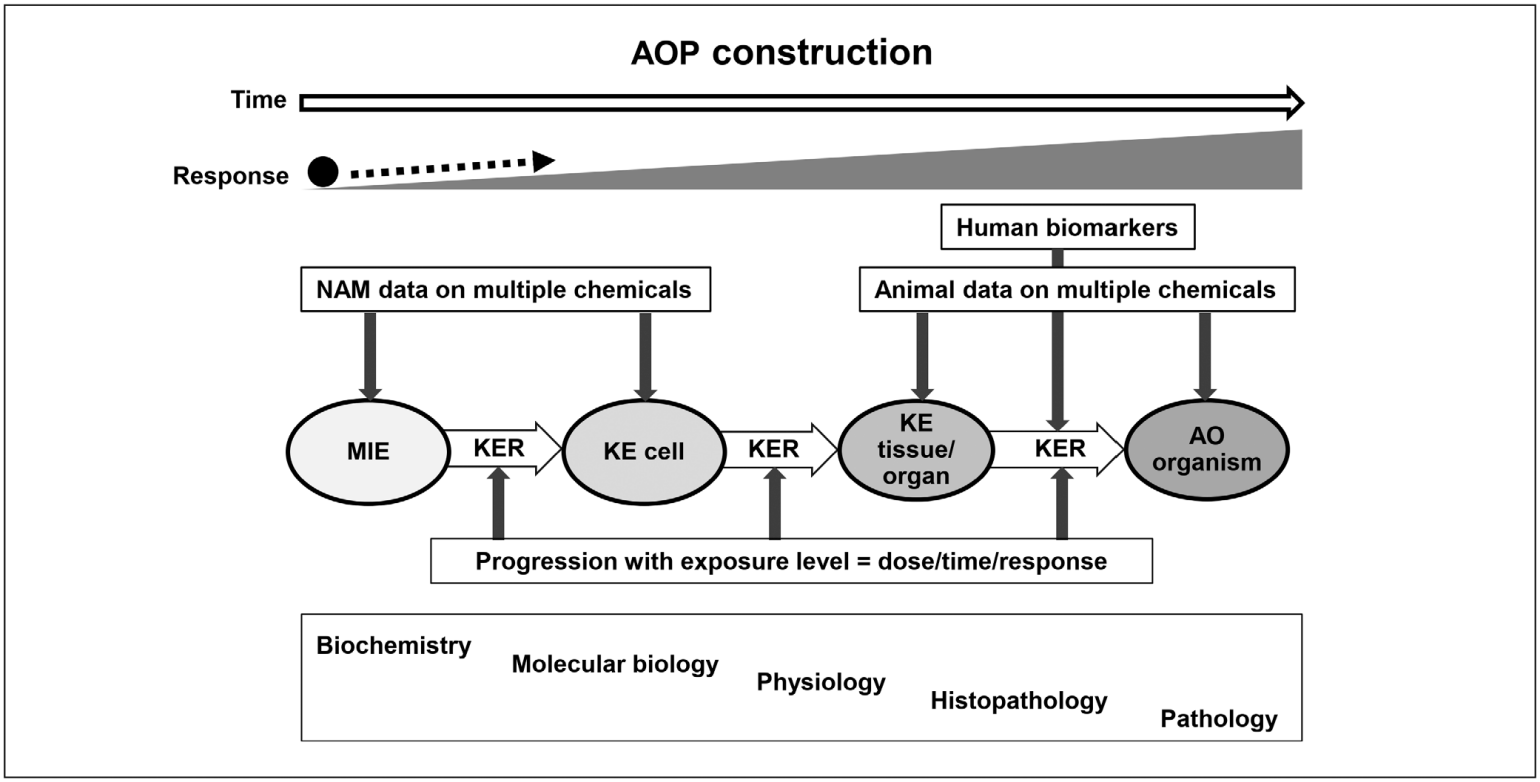
Construction of AOP across the levels of biological organization in relation to dose-response and time-response

The discussion addressed how this could be done. Regarding the construction of an AOP, the importance of reflecting the dose- and time-dependence was stressed, which is a pivotal determinant of the progression of perturbations along the pathway. NAM data on multiple chemicals that allow observations of common responses can be helpful for the identification of early AOP events, particularly the MIEs. The later AOP KEs, which occur at higher levels of organization, can be informed by (histo-) pathological findings in animals and humans observed across multiple chemicals (Fig. 4).

**Fig. 5:**
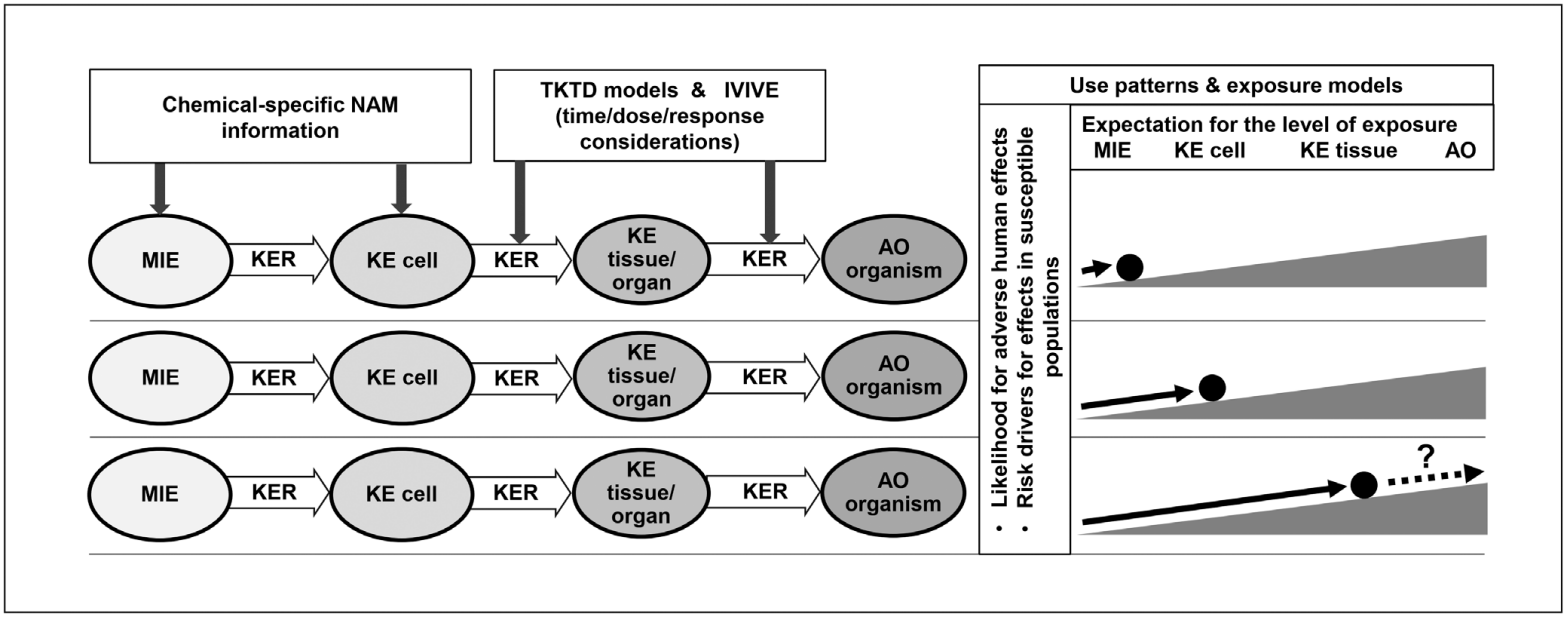
The use of NAM in AOP-based chemical risk assessment AO, adverse outcome; TKTD, toxicokinetic-toxicodynamic; IVIVE, *in vitro* to *in vivo* extrapolation; grey triangle, (increasing) exposure leading to a response; black dot, NAM-evidence supported response; ?, indicator of the uncertainty related to extrapolating the NAM-evidence supported response to an AO

When an AOP is used for risk assessment, the information can be integrated based on actual measurements (e.g., *in vitro* data) or extrapolations from the KERs (e.g., *in vitro* to *in vivo* extrapolation). The dose and time relationships should be examined for concordance. A low dose may produce effects on a MIE, which may or may not be followed by responses at higher levels. The progression in the AOP, with dose and time, can be confirmed by integrating the observed dose-response within the available information on the KERs. NAM may allow for direct measurements at the cellular and tissue levels and estimations on the likelihood of adverse effects for each point in the NAM dose-response relationships. Consequently, NAMs can provide actual evidence on effects up to cell/tissue level, including quantitative information on the KERs, identifying under which exposure conditions these KE responses are expected. At the levels of more complex biological organization, in particular the KERs connecting NAM-based observed effects with adverse endpoints, assumptions and extrapolations based on the available knowledge in a WoE approach are required. As part of the risk assessment process, the available evidence and the uncertainty related to those assumptions and extrapolations should be clearly described. This is indicated by the “?” in Figure 5, representing the risk characterization phase when the likelihood and magnitude of possible adverse effects and the associated uncertainty are considered. Such new frameworks probably require adapted or new approaches to certainty assessment, e.g., regarding biological plausibility.

The discussion on assessing and integrating certainty in AOP-based assessment frameworks based on NAM evidence focused first on the uncertainty related to the AOP and related to NAM. According to the guidance of the OECD for developing and assessing AOP (OECD, 2018), which uses the term confidence, the level of confidence is determined using a categorical rating by assessing the biological plausibility of the KERs according to the modified Bradford-Hill considerations (Meek et al., 2014) and the respective empirical support, including quantitative understanding, as well as the evidence related to the essentiality of KEs. In this regard, it was one of the main conclusions of De Vries et al. (2021) that current evidence assessment domains used within the GRADE framework would be sufficient for the evidence assessment within AOPs, suggesting an attempt to converge the two approaches. Once an AOP is established, with the levels of confidence assessed, application of the AOP to chemical risk assessment requires a standardized approach to informing the KEs and KERs of the AOP with NAM evidence. Regardless of whether existing NAM approaches are used or adapted or whether new ones are developed, the confidence in each individual NAM evidence generated will be driven by how well the NAM models the target KE, and how fit-for-purpose and how reliable, e.g., in terms of precision, reproducibility, and applicability domain, these are. Assessing and measuring that confidence in a transparent and harmonized way was identified as a major challenge before the integration of the evidence as a function of the various confidence-driving aspects as outlined here can even be tackled.

Specific factors to be considered in the certainty assessment include:

- the time to effect and the biological adaptation and recovery, which were considered potentially chemical-agnostic,
- the chemical-specific absorption, distribution, metabolism, and excretion and PBPK modeling
- the variability in the biological response, e.g., in data used for modelling or NAM data used to inform an AOP.

Finally, the potential applicability of the EtD framework to chemical risk assessment was discussed. First, the differences in the decision-making context were pointed out. Chemical assessment frameworks are embedded in regulations, which pre-determine the type of problems to be addressed, whereas the problems intended to be addressed by the EtD framework and the decision-making context are usually not regulated as rigidly. Second, the EtD criteria of Table 2 were reviewed in a chemical risk assessment context. While some were considered to be readily applicable to assessment of risks, such as the problem formulation, the severity of undesirable or adverse effects, certainty assessment, and also feasibility, others were considered to be relevant for the management of risks, e.g., values, balance, resource use, and equity. However, it was agreed that a more thorough discussion, going beyond the scope of this workshop, would be needed, for example, to consider how some criteria could be implemented into risk assessment to better inform risk management (e.g., application of findings across populations).

## 7 Summary and conclusion

Thomas Hartung and Kris Thayer briefly summarized the workshop from their individual perspectives. Thomas Hartung identified four main take-home messages:

- AOP and SR have in common that they are approaches to organize and summarize evidence, facing the same problems, i.e., large amounts of evidence of different types (streams) and of varying quality. It needs to be kept in mind, however, that SR address narrow questions, while AOP are broad in scope.
- Evidence-based approaches can potentially be useful in the context of AOP. Backing up biological plausibility with systematically reviewed evidence has the potential to increase confidence. In particular, KERs could be addressed. As addressing the biological complexity of an AOP would require a number of targeted reviews, which appears to be impractical, (semi-)automated evidence maps are the more promising evidence-based tool. Automation would also facilitate updating AOPs with the latest evidence.
- An efficient discussion on the use of evidence-based methods in constructing and assessing mechanism-focused chemical assessment approaches requires mutual understanding of the terminology used. A controlled vocabulary, e.g., defined via an agreed glossary, would be extremely beneficial in this regard. In addition, controlled vocabularies that can be mapped onto biological ontologies would make information much more accessible, enabling the use of automated retrieval and assessment.
- The usefulness of the EtD framework in the context of chemical risk assessment and management should be explored in more detail.

Kris Thayer focused in her resumé on how certainty can be assessed in evidence frameworks and how this relates to the (modified) Bradford-Hill considerations. Applying the concept from the GRADE Working Group approach to assessing the confidence in a body of evidence, initial confidence in evidence relevant for chemical risk assessment can basically be related to the experimental study design. Determination of the initial level of certainty is currently receiving considerable attention, particularly for the various types of NAM data. The level of certainty is then increased or decreased based on the systematic evaluation of eight (or more) factors. Factors potentially decreasing certainty are, e.g., risk-of-bias, indirectness, inconsistency, and publication bias. Factors potentially increasing certainty are, e.g., large effects and dose-response. This approach is very similar to the Bradford-Hill considerations, as demonstrated by Schünemann et al. (2011). However, the GRADE approach has operationalized the assessment, which facilitates the application and ensures consistency, i.e., reduces the influence of subjectivity in the assessment. In addition, it addresses factors, in particular publication bias, that have been identified as relevant only more recently.

In conclusion, several recommendations emerged from the workshop:

1. Relating biological knowledge to an AOP can be viewed as a process amenable to SR, moving from observed associations between biological events to identifying those event-event relationships for which we have enough certainty to incorporate them into human health risk assessments.
2. Application of systematic review methods to AOP development is challenging due to different research contexts, vocabulary, and reasoning processes employed in the two approaches; considerable work is required to interpret and translate processes and vocabulary if the lessons from one are to be fully applied to the other.
3. The rigor and transparency inherent to evidence-based and systematic approaches can contribute to building stakeholder trust for implementation of NAM evidence and AOP into chemical risk assessment.
4. The iterative nature of AOP development requires a combination of evidence maps to define the KE and overall structure of the AOP and SR to perform the evidence evaluation for each KER. Methods for allowing semantic mapping and search expansion during the literature inventory or evidence mapping step(s) are essential because the full breadth of the literature needed is rarely known *a priori*.
5. The time required for a full evidence map and SR covering all KERs within an AOP is prohibitive. The use of text-mining tools to facilitate the reviews is highly recommended. AOP developers should focus their efforts on those KERs that are most relevant for the current purpose. AOP documentation in a public knowledgebase allows the evaluation of additional KERs in a resource-dependent manner.
6. Defining a PECO statement for AOP development is problematic, since a single AOP would include multiple SRs covering the individual KERs. Therefore, up-front problem formulation (including the assessment of resources and goals) is of utmost importance in developing and establishing an approach (including systematic methodologies) for interrogating any AOP pathway or its components.
7. NAM and AOP-based chemical risk assessment will require a change in chemical risk assessment methodology, shifting the focus from AOs assessed via animal experiments to AOP-structured information on pathway perturbations.
8. Harmonized and transparent assessment and measurement of the confidence in the AOP and the NAM populating it was identified as a challenge and a prerequisite for the integration of the evidence as a function of the various confidence-driving aspects.
9. The potential application of the EtD framework to chemical risk assessment should be explored in more detail.

## Figures and Tables

**Tab. 1:** Preliminary mapping of the concepts of the modified Bradford Hill considerations (OECD, 2018) to the concepts of certainty in the evidence in the GRADE approach

Assessing certainty in AOPs using the modified Bradford Hill considerations		Relationship to GRADE
Modified Bradford Hill considerations	Criteria for high confidence	
Biological plausibility (Is there a mechanistic relationship between an upstream and downstream KE which is consistent with established biological knowledge?)	Extensive understanding of the KER based on extensive previous documentation and broad acceptance (e.g., mutation leading to tumors), i.e., an established mechanistic basis	Biological plausibility is not explicitly part of GRADE, being operationalized under the indirectness domain. Further development of GRADE to respond to considerations around biological plausibility is currently being considered (Whaley et al., 2022).
Essentiality (Are downstream KE and/or the adverse outcome prevented if an upstream KE is blocked?)	Direct evidence from specifically designed experimental studies illustrating essentiality for at least one of the important KE.	Testing of counterfactuals is not part of GRADE. It might become a necessary part of the evidence for developing knowledge of mechanisms. “Essentiality” might be a question that is addressed via a direct SR question rather than as a certainty criterion.
Empirical support	Dependent change in related events following exposure to stressors, accompanied by evidence for temporal, dose-response, and incidence concordance. The more evidence of each, the greater the confidence in the empirical support.	Dose-response is a distinct domain within GRADE. Temporality is accommodated under risk of bias assessment (though risk of bias is not currently part of AOP development). The possibility of increased certainty from seeing more of an upstream event than a downstream event may be additional considerations for the GRADE domains.

**Tab. 2:** Criteria of the GRADE EtD framework

**Criteria**	
Problem	Is the problem a priority?
Desirable and undesirable effects	How substantial are the desirable/undesirable anticipated effects?
Certainty	What is the overall certainty of the evidence of effects?
Values	Is there important uncertainty about or variability in how much people value the main outcomes?
Balance	Does the balance between desirable and undesirable effects favor the option or the comparison?
Resource use	How large are the resource requirements (costs)? What is the certainty of the evidence of resource requirements? Does the cost effectiveness of the option favor the option or the comparison?
Equity	What would be the impact on health equities?
Acceptability	Is the option acceptable to key stakeholders?
Feasibility	Is the option feasible to implement?

## Box 1: Stages in considering the extent (weight) of evidence in hazard characterization

- Defining the question
- Identifying and organizing data (“categorizing”)
- Selecting data (regarding relevance and reliability)
- Reviewing pertinent study considerations (based on predefined (contextually relevant) criteria
- Data synthesis/integration (within/across lines of evidence) →AOP/MoA
- Application to decision-making

## Footnotes

1
https://aopkb.oecd.org

2
https://aopwiki.org

3
www.decide-collaboration.eu

4
https://gradepro.org/

5
https://healthcare-quality.jrc.ec.europa.eu/
